# A Case of Coexistent Sarcoidosis and Tuberculosis: A Diagnostic Dilemma

**DOI:** 10.7759/cureus.37667

**Published:** 2023-04-17

**Authors:** Nader Francis, Mai Khouly, Gangaiah Komala, Sinan Yavuz

**Affiliations:** 1 Pediatric Pulmonology, Al Qassimi Women's and Children's Hospital, Sharjah, ARE; 2 Paediatrics, Al Qassimi Women's and Children's Hospital, Sharjah, ARE; 3 Pathology Laboratory, Al Qassimi Hospital Women's and Children's Hospital, Sharjah, ARE; 4 Paediatrics Pulmonology, Al Qassimi Women's and Children's Hospital, Sharjah, ARE

**Keywords:** active pulmonary tuberculosis, pediatrics, caseating granulomas, pulmonary sarcoidosis, tuberculosis

## Abstract

Sarcoidosis and tuberculosis (TB) are chronic granulomatous diseases with similar radiological, clinical, and histopathological presentations. Although rare, both conditions can coexist together. Case reports of concomitant incidence have been published in the literature. The classic manifestations of both diseases overlap, making it difficult for clinicians to reach a final diagnosis. While TB is responsible for the majority of necrotizing granuloma cases, necrotizing sarcoidosis should be considered a possible diagnosis, especially in the absence of mycobacterial antigen isolation or when a remarkable improvement isn’t achieved after administering anti-tb medications.

We report a rare case of a 12-year-old female exhibiting an atypical form of the granulomatous disease (concomitant incidence of tuberculosis and sarcoidosis), who presented with respiratory distress, cough, fever, weight loss, and generalized fatigue that was initially diagnosed as Tuberculosis which was supported by radiological and biological findings. Initially, the patient had shown some clinical improvement with anti-tubercular treatment, but nonetheless, she experienced progressively increasing mediastinal lymphadenopathy. Subsequently, she developed new granulomatous skin findings. Further investigations supported the diagnosis of coexisting sarcoidosis.

## Introduction

Sarcoidosis and tuberculosis (TB) are chronic granulomatous diseases with similar radiological, clinical, and histopathological presentations. Although rarely both conditions coexist together, case reports of concomitant incidence of these two conditions have been published in the literature [1]. Given the overlapping manifestations, this poses a diagnostic challenge to clinicians to diagnose them individually and treat them accordingly. 

Sarcoidosis is an immunological multisystem granulomatous disorder of unknown etiology, characterized by the presence of noncaseating granulomas in different organs. Although a majority of patients affected by sarcoidosis have lung manifestations, 10-30% of people affected have extra-pulmonary manifestations in the skin, liver, spleen, lymph nodes, heart, and nervous system [2].

TB is an infectious disease caused by Mycobacterium tuberculosis (MTB) and is defined histopathologically by granulomas with caseous necrosis [3]. However, some TB granulomas are non-caseating, and even the distinction between caseation and noncaseating is not absolute [4]. Early diagnosis and distinguishing between the two entities is vital to prevent irreversible damage to the lungs, particularly in treatment-responsive interstitial lung disease (ILD) such as sarcoidosis [5]. Diagnostic modalities such as the Mantoux test, serology (QuantiFERON), high-resolution computed tomography (CT), transbronchial lymph node, and lung biopsies all aid in reaching the diagnosis. 

## Case presentation

**Figure 1:**
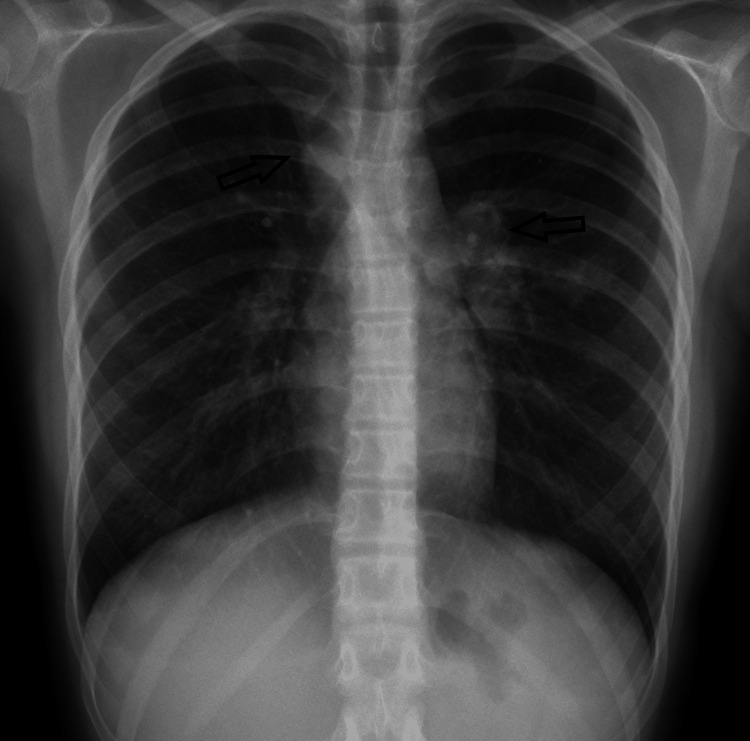
Chest x-ray revealed right superior mediastinal opacity in the right paratracheal stripe. Prominent left hilar and left parenchymal non-homogeneous opacity pointed at with the arrows.

We present a case of a 12-year-old girl with a history of intermittent fever, weight loss of 5 kgs, and cough for 27 days, associated with weakness, muscle pain in her lower extremities, and occasionally mild chest pain. Fever reaching 40 C mainly at night associated with chills and sweating. The cough was productive and associated with dyspnea. Clinical examination was unremarkable. Chest x-ray showed a right superior mediastinal opacity along the right paratracheal stripe and prominent left hilar and left parenchymal non-homogeneous opacities pointed at with the arrows (Figure 1).

A CT scan of the chest showed the left lower lobe superior bronchopulmonary segment with a patch of consolidation with irregular margins and perilesional fibrosis. A few small enlarged mediastinal lymph nodes were noted. Features are suggestive of pulmonary tuberculosis involving the left lower lobe as described (CT image is unavailable due to technical issues).

Investigations showed a high erythrocyte sedimentation rate (ESR) count, with positive tuberculin skin testing (PPD) (20 mm) and a positive Gamma interferon TB antigen. Induced Sputum morning samples taken on three consecutive days were all negative for acid-fast bacilli (AFB). So we decided to go for bronchoscopy, which showed a mildly enlarged carinal angle and a mild left main bronchus stenosis (around 20-30%), with normal mucosa, and no granulomas nor secretions were seen. Bronchoalveolar lavage (BAL) for acid-fast bacilli smear (AFB) was negative, polymerase chain reaction (PCR) MTB came positive, and GeneXpert detected TB, sensitive to rifampicin. Hence an initial diagnosis of TB was made, and the patient was started on anti-TB medications with proper dosages (isoniazid, rifampin, pyrazinamide, and ethambutol for two months, isoniazid plus rifampicin for four months). The fever settled after nine days of starting treatment. 

**Figure 2:**
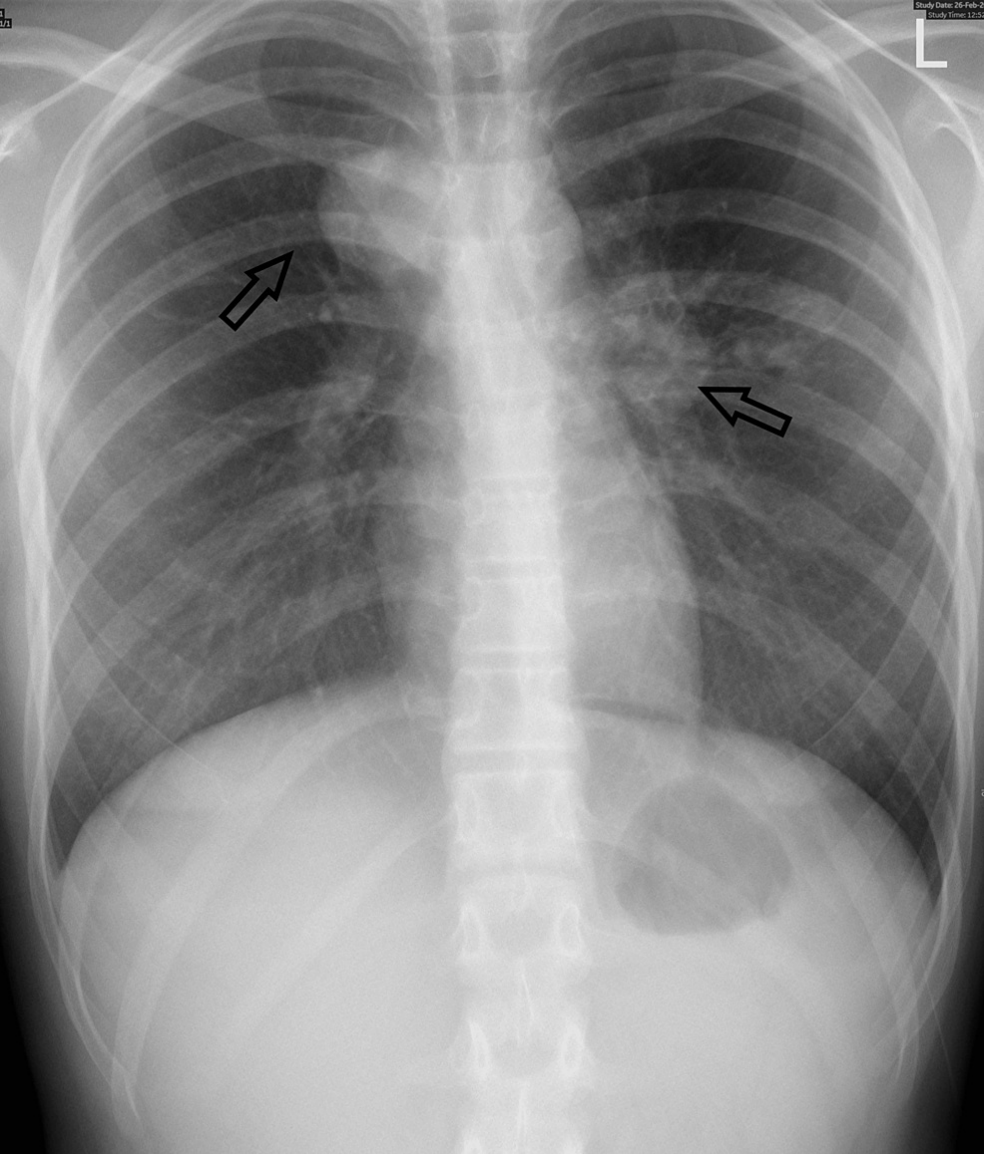
Repeated chest x-ray showed an increase in the volume of the hilar adenopathy and enlargement of the mediastinum (black arrows).

One month after starting treatment, the patient came for a follow-up (compliant with anti-TB medicines). The fever pattern improved. However, she was still complaining of cough, chest pain, and difficulty breathing. Spirometry was normal. Repeated chest x-ray showed an increase in the volume of the hilar adenopathy and enlargement of the mediastinum (Figure 2).

A new CT chest with contrast showed findings of extensive enlarged and amalgamated mediastinal lymph nodes involving all the mediastinal groups. Multiple patchy consolidations with irregular margins involving the superior segment of the left lower lobe are associated with perilesional fibrosis and traction bronchiectasis. The differential diagnosis at this time consisted of Lymphoma, Mediastinal tumor, sarcoidosis, or possibly a paradoxical reaction to TB treatment. Angiotensin-converting enzyme (ACE) level was done at another facility which was reported to be normal, and urinary calcium level was normal (1.5 mmol/l). Afterward, the CT scan we decided to go for a biopsy to exclude the other differentials

A cervical lymph node biopsy was done in their home country (Pakistan) as it was the patient’s preference. Which confirmed the diagnosis of TB (scanty AFB, PCR positive to MBT and sensitive to Rifampicin, culture negative for eight weeks). The decision was made to continue anti-TB treatment. After coming back, symptoms were persistent despite being compliant with anti-TB medications.

**Figure 3:**
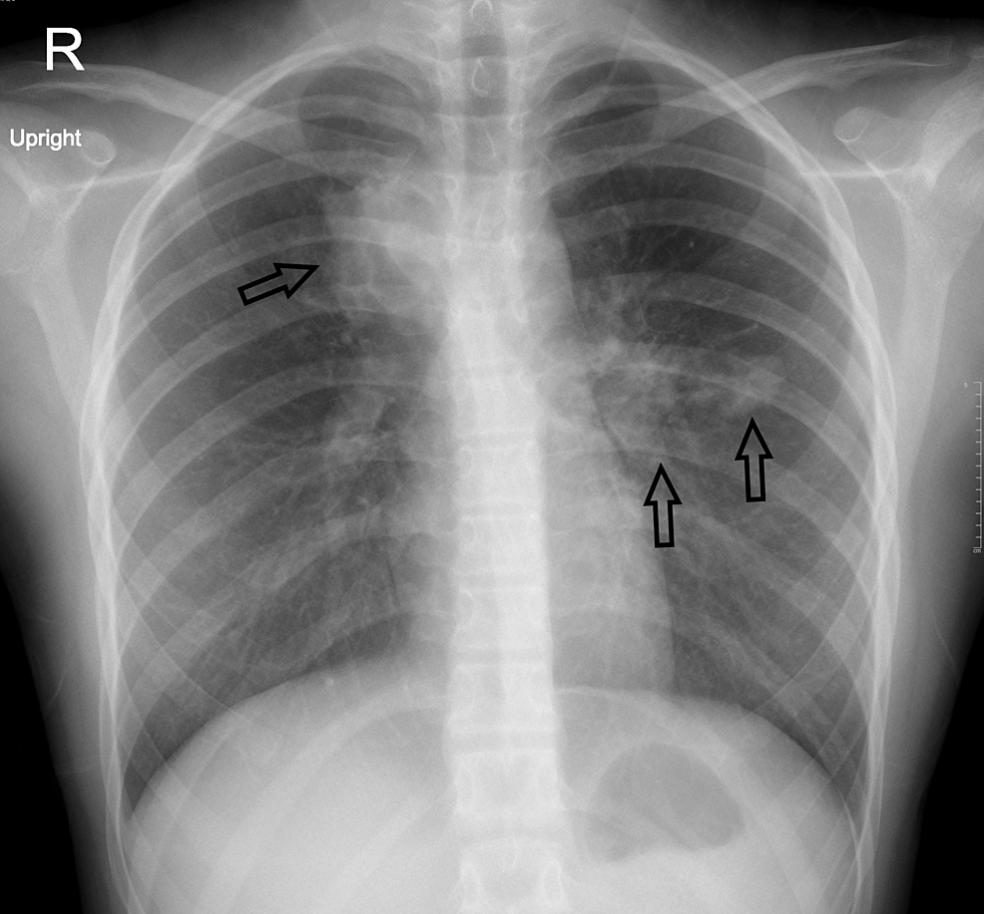
Repeated chest x-ray showing an increase in the mediastinal mass (black arrows).

After three months, the patient was still having chest pain, chest tightness, cough, fatigue, and leg pain but no fever. We discovered a new granulomatous skin lesion (a small subcutaneous nodule appeared on the left side of the anterior abdominal wall, measuring 1x1 cm, well-defined, non-tender). Repeated chest x-ray showing an increase in the mediastinal mass (Figure 3).

**Figure 4:**
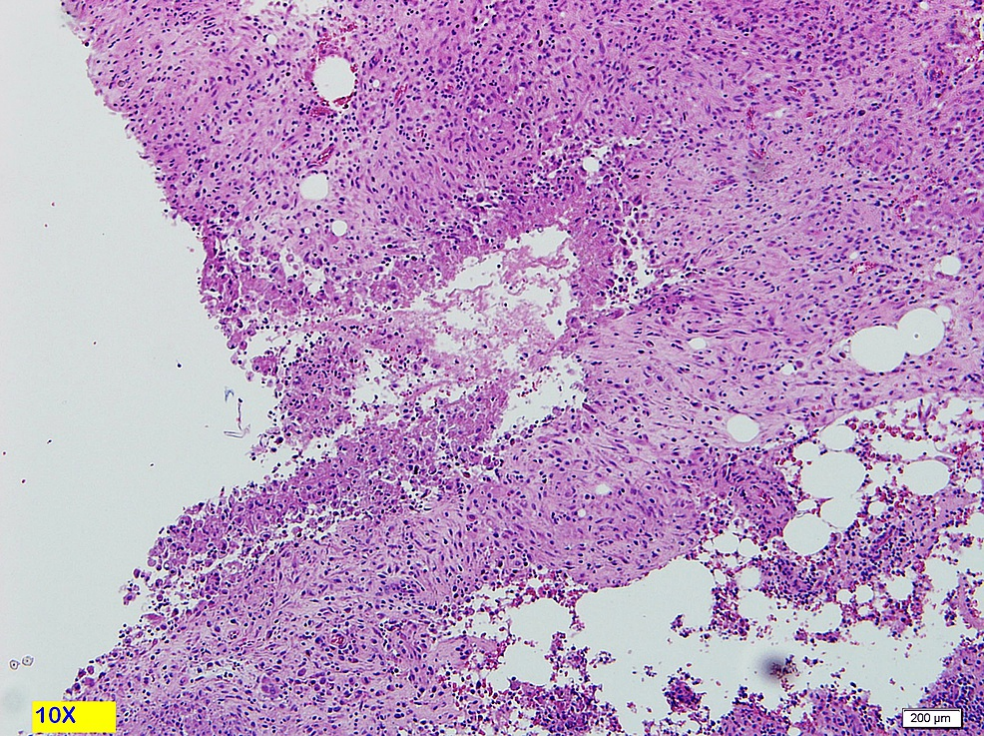
10X magnification. H&E stain. Showed subcutaneous necrotizing epithelioid granulomas.

**Figure 5:**
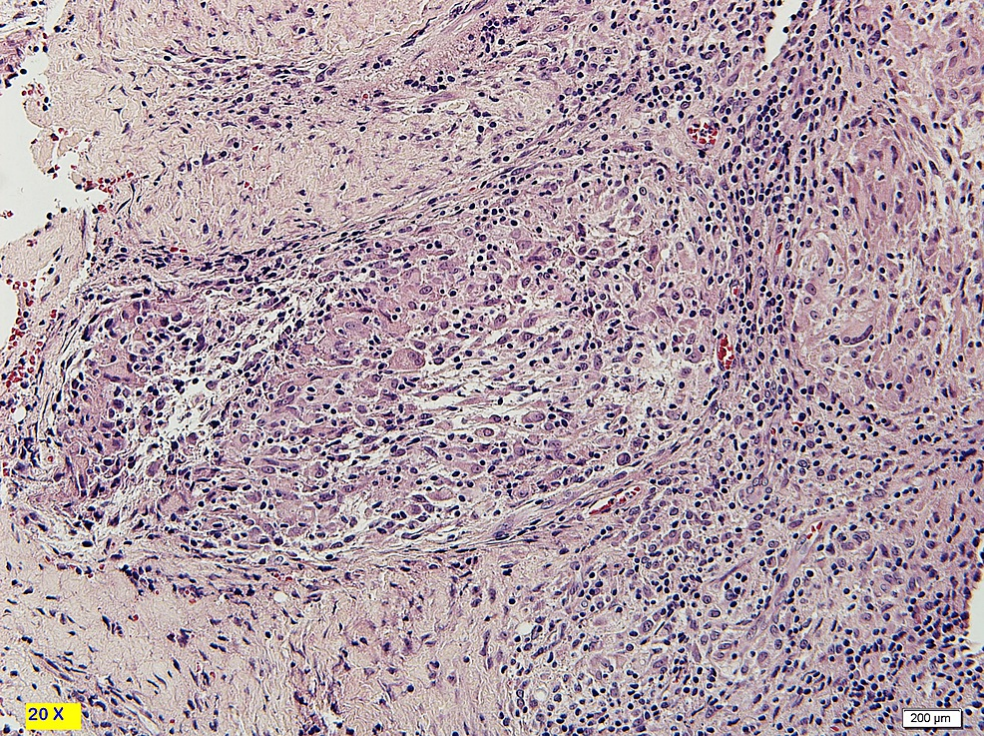
20X magnification, H&E stain. Epithelioid and histiocytic granulomas with Langhans giant cells. There is lymphocyte and plasma cell cuff around the granulomas.

**Figure 6:**
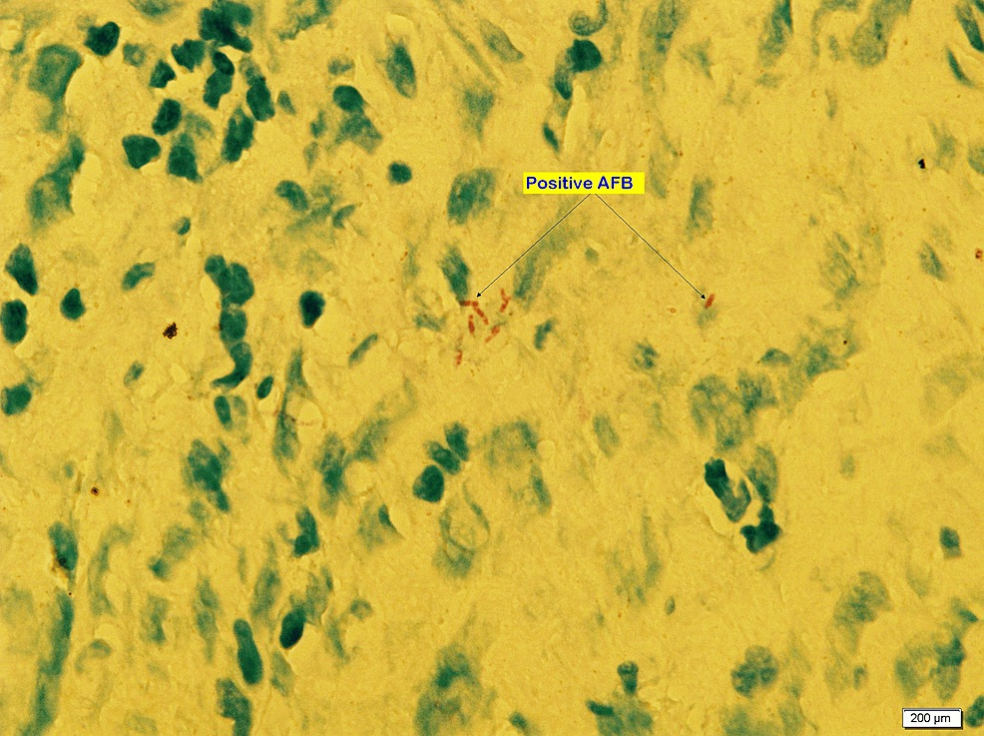
100X magnification, ZN stain. Show positive pink rods of acid-fast bacilli (AFB) within granulomas.

**Figure 7:**
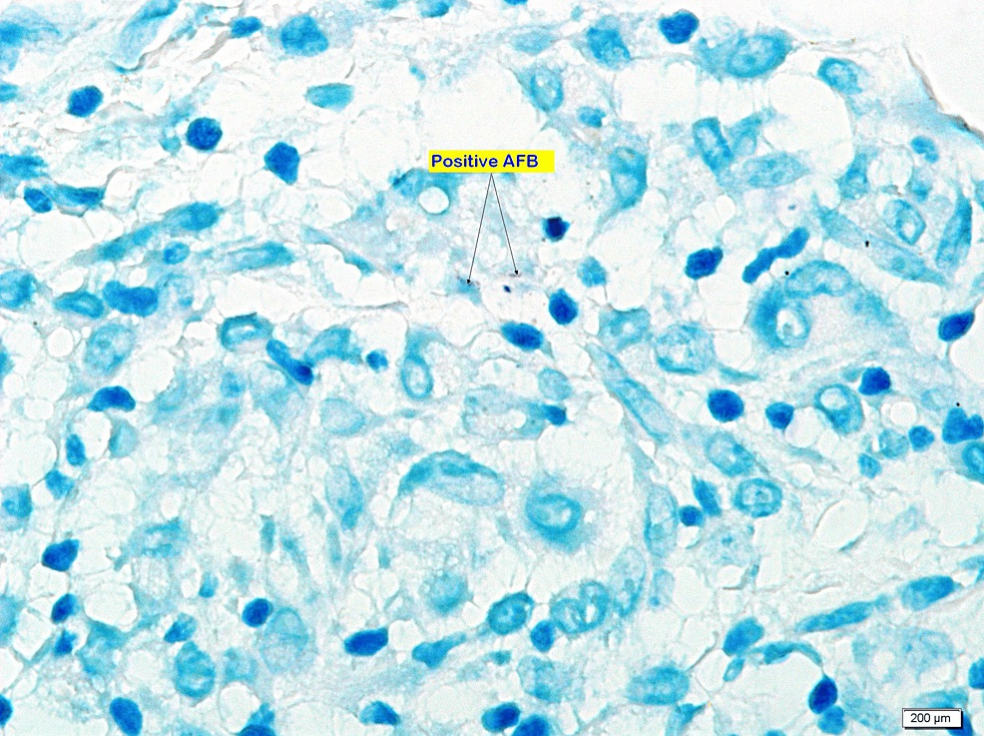
100X magnification, ZN stain. Show positive pink rods of acid-fast bacilli (AFB) within granulomas.

The enlarged lymph node and abdominal wall lesion prompted biopsy from the abdominal mass and the cervical lymph node. Abdominal mass biopsy showed a lesion with epithelioid granulomatous inflammation consistent with Mycobacterial infection. Epithelioid and histiocytic granulomas with Langerhans giant cells with lymphocyte and plasma cell cuff around the granulomas also showed positive AFB smear, Mycobacterium Bacilli stained in red (Figures 4-7).

A team discussion was carried out, based on the histopathology that was going with tuberculosis (PCR positive to TB, AFB smear positive, with negative cultures), the clinical course, the newly developed granulomatous skin lesion, the growing mediastinal mass and no improvement of symptoms despite having good compliance to anti-TB medications, that the most probable diagnosis of our patient was favoring sarcoidosis.

**Figure 8:**
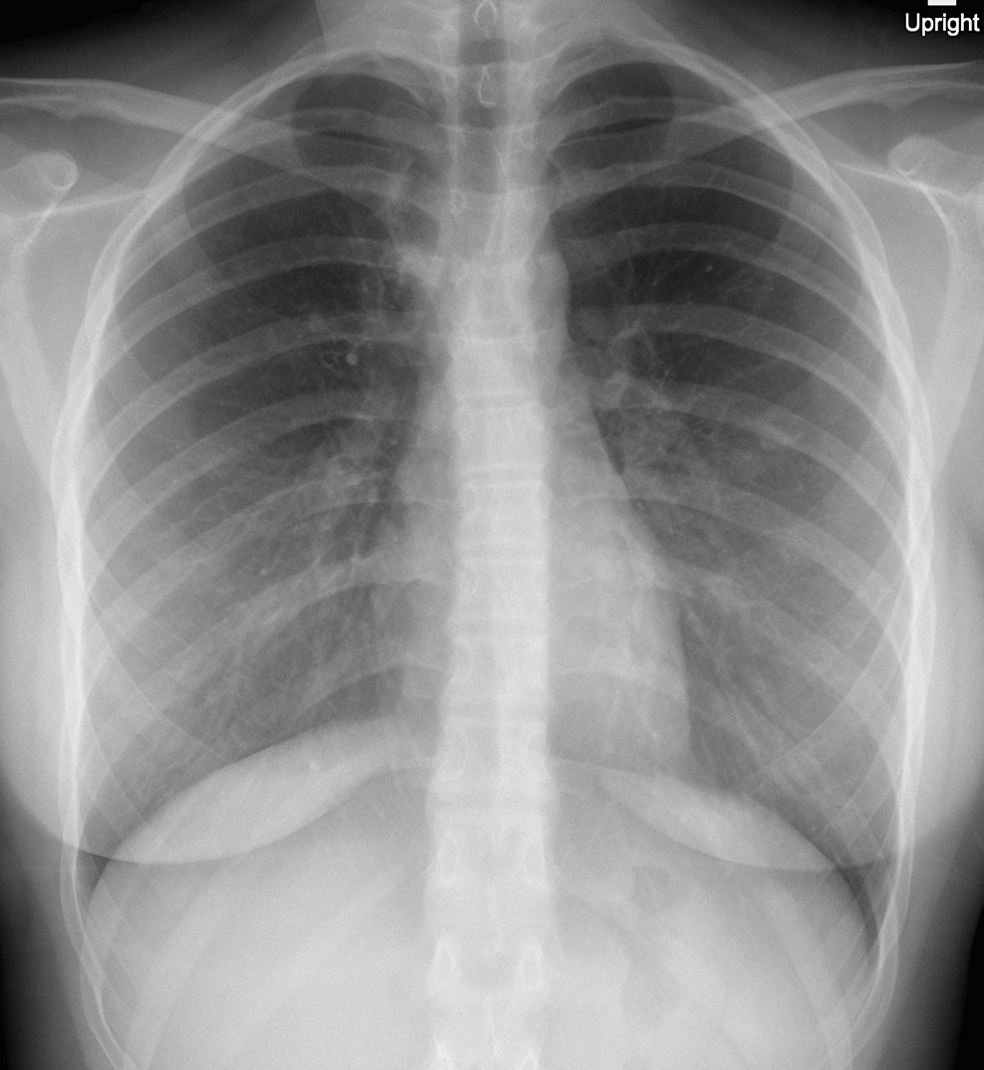
Chest X-ray showed improvement, with a significant reduction of the hilar mediastinal mass and resolution of parenchymal nodular infiltrates.

A decision of starting Prednisolone 1mg/kg/day as a treatment for sarcoidosis was carried out. After the initiation of treatment, the patient was feeling better, the cough had been remitted, with no respiratory distress or any feeling of chest tightness. Spirometry demonstrated normal lung function, and radiographic appearances showed improvement, with a significant reduction of the hilar mediastinal mass and resolution of parenchymal nodular infiltrates. With regular follow-ups, patient was improving, having a good response to treatment (Figure 8).

On serial follow-ups for three years, symptoms completely resolved with no relapse, and serial imaging remained reassuring.

## Discussion

Tuberculosis and sarcoidosis are chronic granulomatous diseases that present with clinical, radiological, and histological similarities. Sarcoidosis is an autoimmune disease with multisystem involvement, resulting in noncaseating cell granulomas. As opposed to TB being an infective disease caused by MTB resulting in caseating granulomas [4].

The diagnosis of either disease depends on the presence of either caseating or non-caseating granulomas in a biopsy specimen and the documentation of the presence of Mycobacterium tuberculosis (AFB, PCR, culture) in case of tuberculosis. However, noncaseating granulomas may be present with MTB, and sarcoidosis granulomas have necrotic features in up to 1/3 of cases. There is an overlap in the presence of caseation between both diseases, making it challenging to differentiate between the two entities based solely on the presence or absence of necrosis in a granulomatous lesion [5]. 

Several studies have proposed that mycobacteria may act as a triggering antigen and elicit an immunological response and eventually induce sarcoidosis, especially in genetically predisposed patients [6]. Therefore, in cases of suspected sarcoidosis, the exclusion of other causes of granulomatous disease such as TB has to be made. As it may be difficult to visualize the caseation in a granuloma, the diagnosis of TB must be made by detecting the causative agent (Mycobacterium tuberculosis, MTB) in an appropriate biological specimen. A direct microscopic examination to identify acid-fast bacilli is usually done but has its limitations and poor sensitivity compared to biomolecular testing. So having a nucleic acid amplification (NAA) test is a good alternative, with higher sensitivity and specificity on respiratory specimens [7]. Detection of MTB DNA in 50%, and non-TB mycobacterial DNA in a further 20% of patients with sarcoidosis, has shown possible connections between the two entities [8] concluding that TB could be a causative factor for sarcoidosis. The coexistence of both diseases is rare but possible.

The mainstay of treatment in sarcoidosis is glucocorticoids, and it is associated with several serious adverse effects. Therefore, treatment is indicated only when symptoms are disabling and/or the granulomatous inflammation is persistently progressive, causing potentially debilitating or life-threatening sequelae [9]. As in our case, the patient had progressive disease deterioration. Hence the reason, steroids were started, which led to the complete resolution and regression of the disease. Although reactivation of tuberculosis after corticosteroid treatment for sarcoidosis is an important concern, given the high prevalence of latent tuberculosis infection worldwide [10]. 

The patient had suggestive symptoms along with a positive tuberculin skin test, PCR TB, and TB serology (QuantiFERON) and radiological findings supporting the suspicion of TB, which gave sufficient information to initiate the anti-TB medications. But with persistent symptoms, and the worsening of the mediastinal lymphadenopathy, and knowing that both diseases could be concomitant [3]. Immunosuppressive medications as a treatment for sarcoidosis had to be considered. The occurrence of both diseases simultaneously posed a diagnostic dilemma and therapeutic challenge. A few other case reports have also shown a diagnostic dilemma when both these conditions concomitantly affected patients [11,12].

## Conclusions

An Infective etiology has to be excluded before assuming the diagnosis of sarcoidosis, and even after visualization of a caseating granuloma in a biopsy specimen, sarcoidosis can’t be excluded if clinically suspected. The decision to start anti-TB medications could be justified; however, if there was no improvement either clinically or radiologically after initiation of anti-TB treatment, other differential diagnoses or the coexistence of sarcoidosis should be considered to start treating for sarcoidosis effectively. Our case report shows the importance of keeping a differential diagnosis of TB and Sarcoidosis overlap in suspected cases such as our patient to keep a low threshold for considering initiation of immunosuppressive therapy to ultimately prevent irreversible damage to the lungs that could occur as a consequence of sarcoidosis.
